# Supplementary material for: GPCR-specific autoantibody signatures are associated with physiological and pathological immune homeostasis
Source: Nat Commun. 2018 Dec 6;9:5224. doi: 10.1038/s41467-018-07598-9 (PMC6283882; doi:10.1038/s41467-018-07598-9)
Supplement: Supplementary file 1 — Supplementary Information [file 41467_2018_7598_MOESM1_ESM.pdf]

**GPCR-specific autoantibody signatures are associated with physiological and pathological immune homeostasis.**

Cabral-Marques et al.

# Supplementary Figure 1

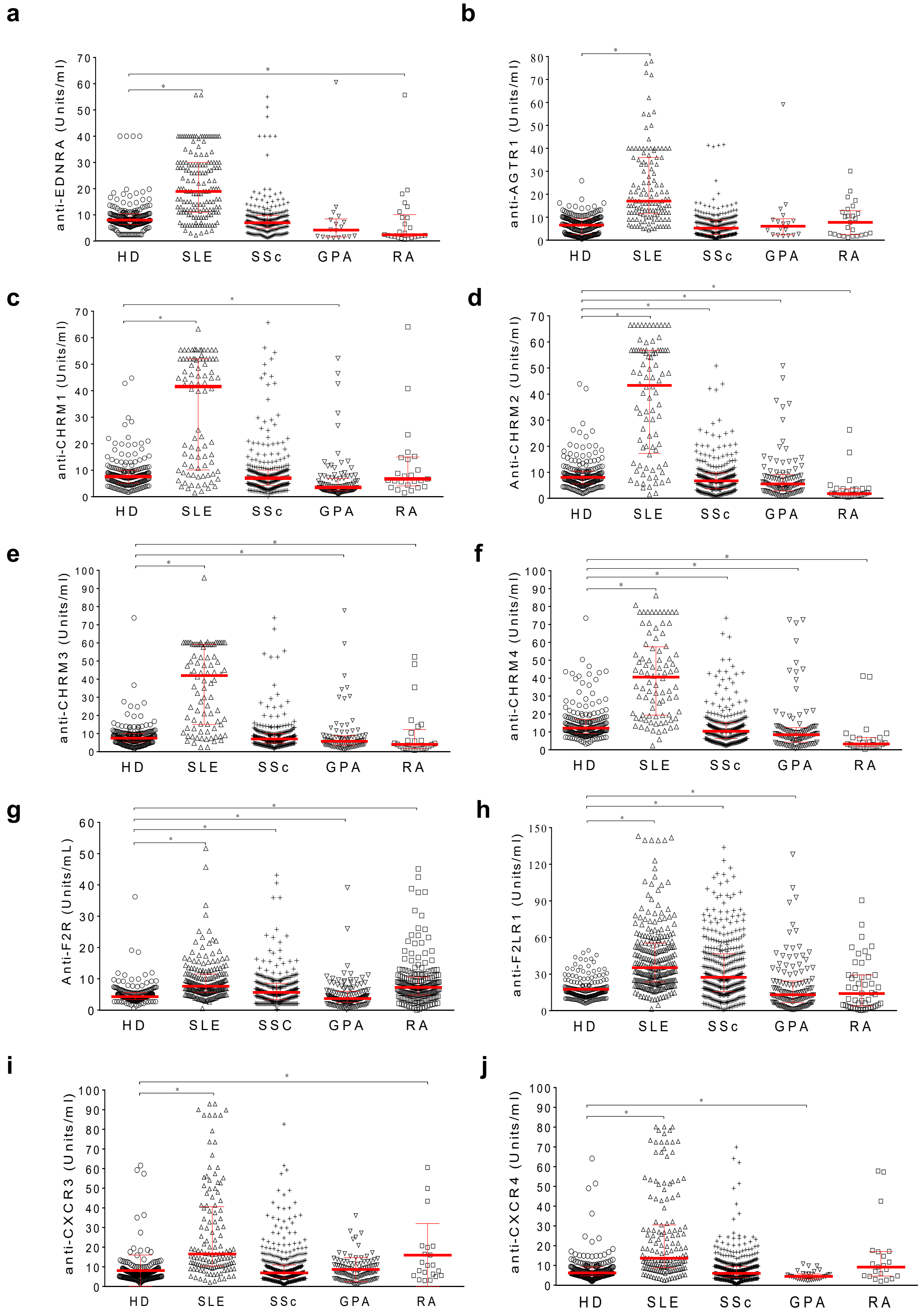


Supplementary Figure 1. Dysregulation of autoantibody concentrations in patients with autoimmune diseases. Graphics show the concentrations of aab directed against A-J) 10 different GPCRs, comparing healthy donors (HD, n=197) to patients with autoimmune diseases. A total of 249 patients with systemic lupus erythematosus (SLE), 379 patients with systemic sclerosis (SSc), 128 patients with granulomatosis with polyangiitis (GPA), and 196 with rheumatoid arthritis (RA) were screened in this phase of the investigation. However, not all patients could be screened for the 10 aab due to sample limitations. The median with interquartile range is shown in red. *, $p<0.05$ (Mann-Whitney test).

## Supplementary Figure 2

a

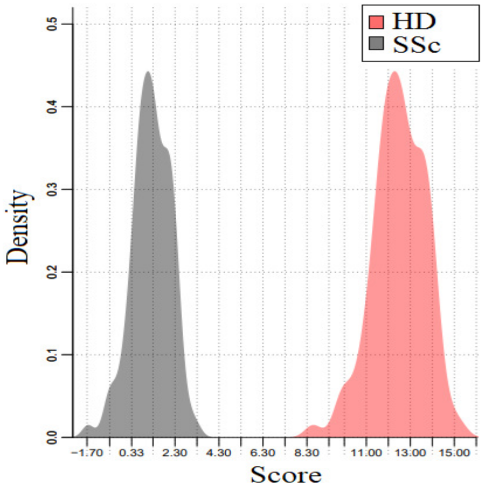


b

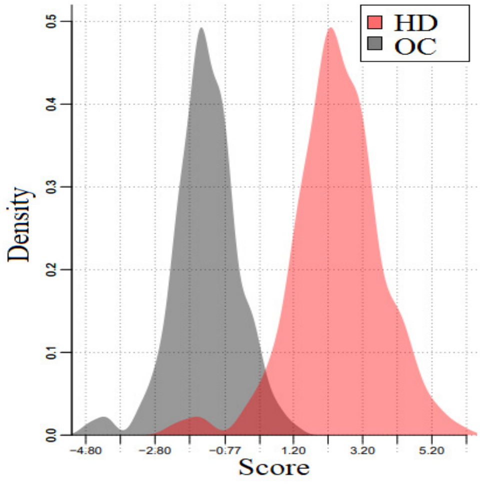


c

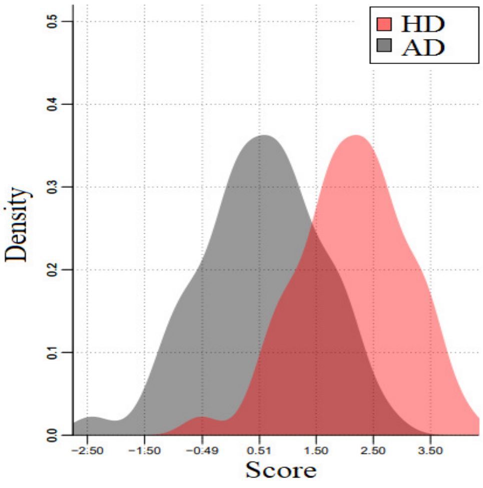


Supplementary Figure 2. Linear discriminant analysis of autoantibody signatures differentiates healthy subjects and patients. Density plots of the linear discriminating scores show the separation between individuals belonging to the disease groups compared with healthy donors (HD). A) HD versus patients with systemic sclerosis (SSc, Supplementary Table 1, cohort 1; Supplementary Table 2, aab dataset 1, B) HD versus patients with ovarian cancer (OC, Supplementary Table 1, cohort 2; Supplementary Table 2, aab dataset 2), and C) HD versus patients with Alzheimer’s disease (AD, Supplementary Table 1, cohort 3; Supplementary Table 2, aab dataset 3).

**Supplementary Figure 3**

**a**

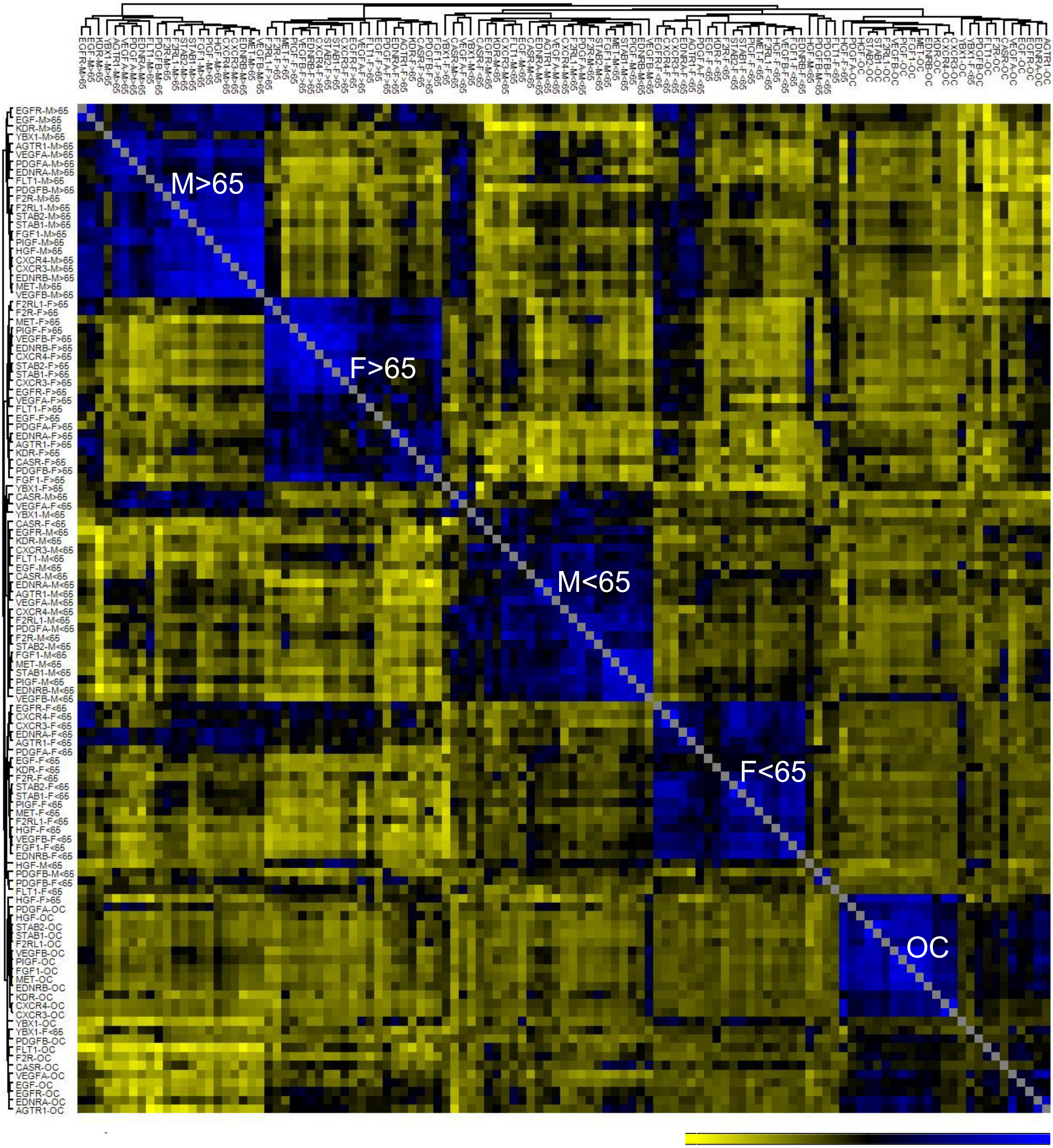

**b**

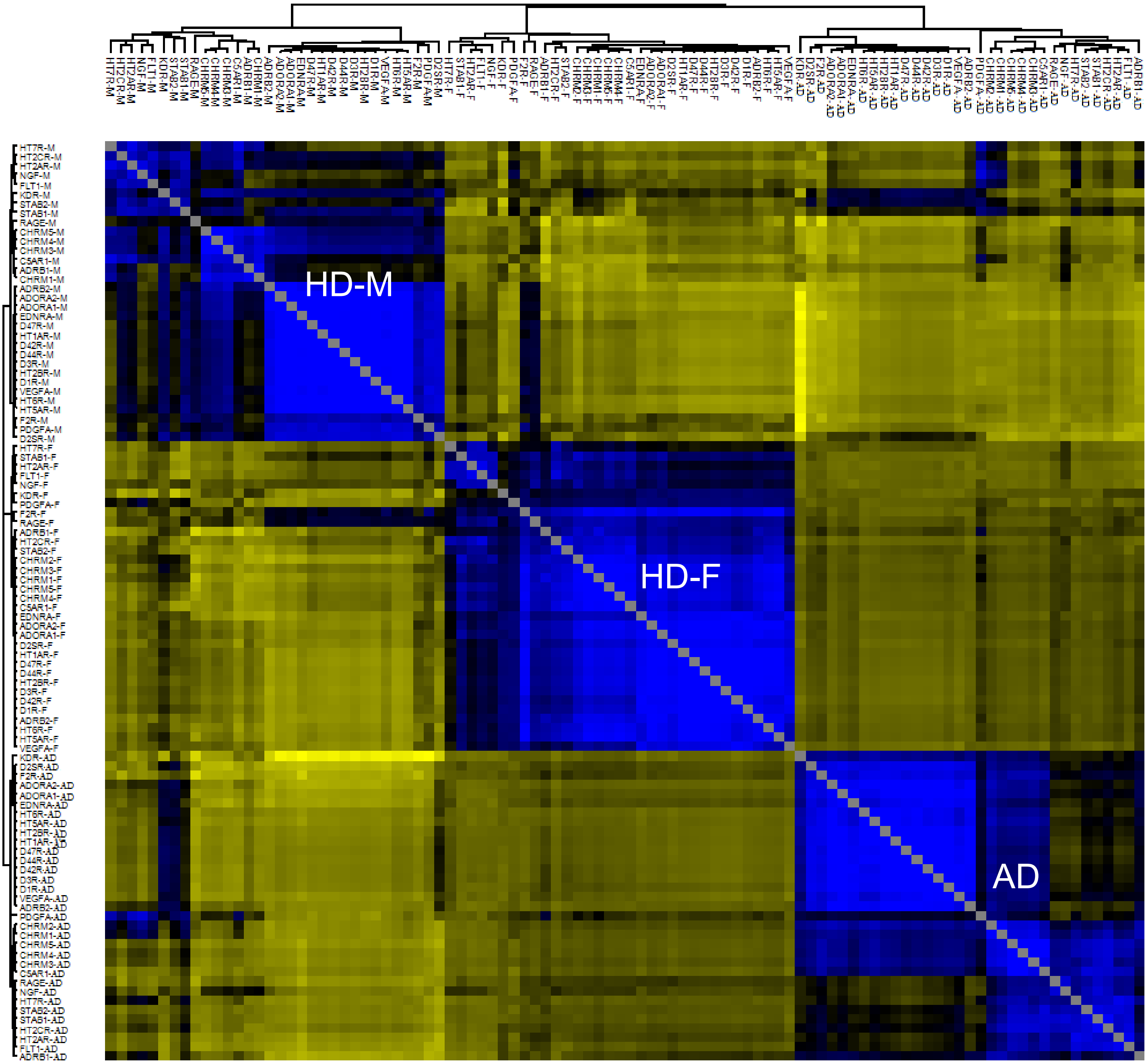

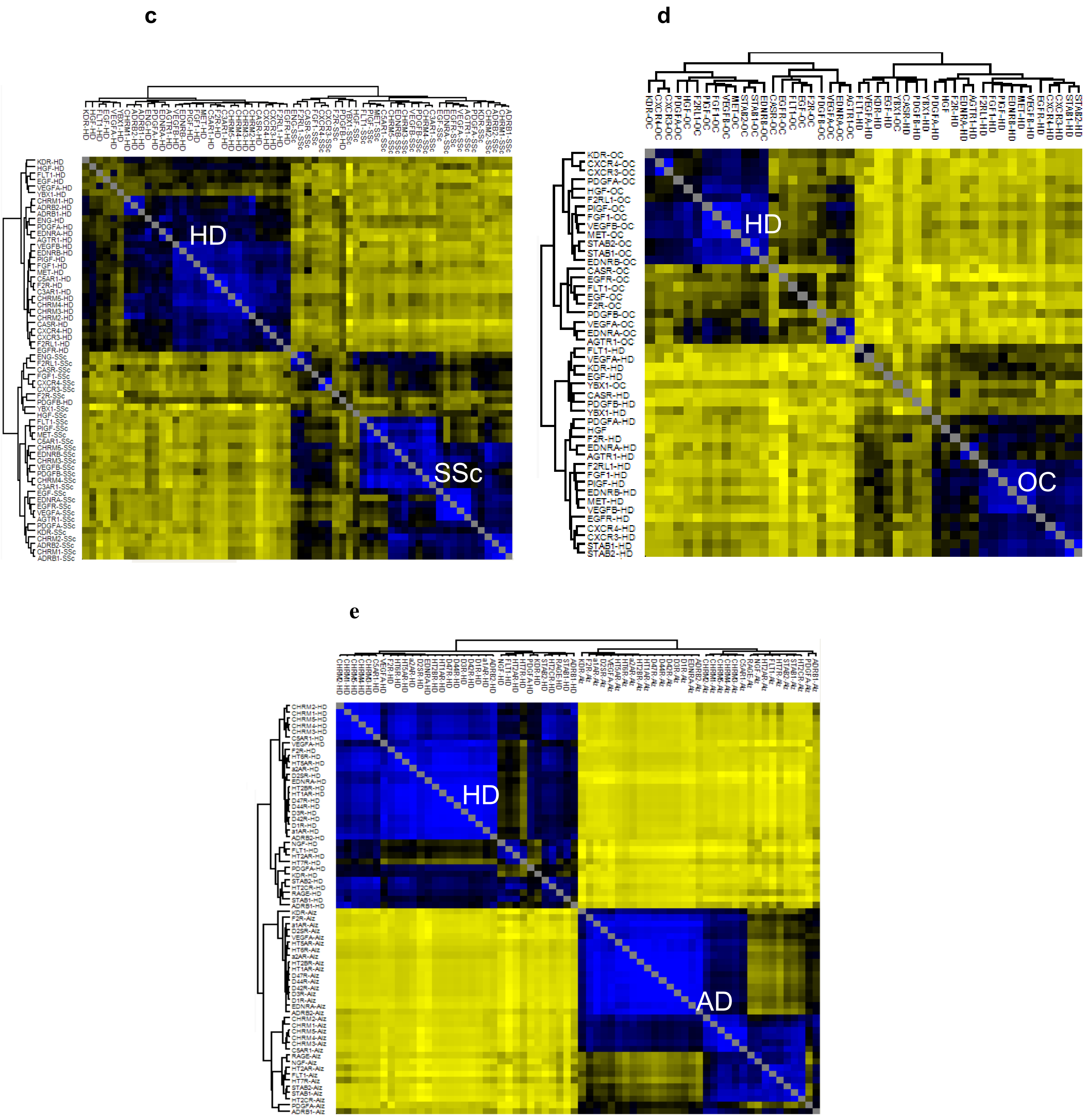


Supplementary Figure 3. Hierarchical clustering analysis reveals autoantibody correlation signatures according to gender, age and diseases. Correlogram matrices display clusters of aab. A) The heatmap displays the clusters of aab correlations from subgroups (females and males aged < and ≥65 years) of healthy donors (HD) versus ovarian cancer (OC; Supplementary Table 1, cohort 2; Supplementary Table 2, aab dataset 2). B) HD in relation to patients with Alzheimer's disease (AD, Supplementary Table 1, cohort 3; Supplementary Table 2, aab dataset 3). The correlation matrices used to perform the hierarchical correlogram of OC and AD are provided as source data. Due to the small number of healthy males <65 years of age (HD cohort 3), we only performed hierarchical clustering analysis of this group according to gender. Supplementary Table 1 provides further details about the HD and patient groups. Analyses of nonsubgrouped C) HD compared with systemic sclerosis (SSc), D) HD versus ovarian cancer (OC), E) and HD in relation to patients with Alzheimer's disease (AD) are shown. Dendrograms on the top and side of the correlation matrix display clusters of correlation between aab. In the heatmap matrix, each small square represents a pairwise correlation between aab. The bar ranging from yellow to blue (A-B: -0.5 to 0.9; C-E: -0.3 to 1) represents negative to positive correlations, respectively.

## Supplementary Figure 4

a

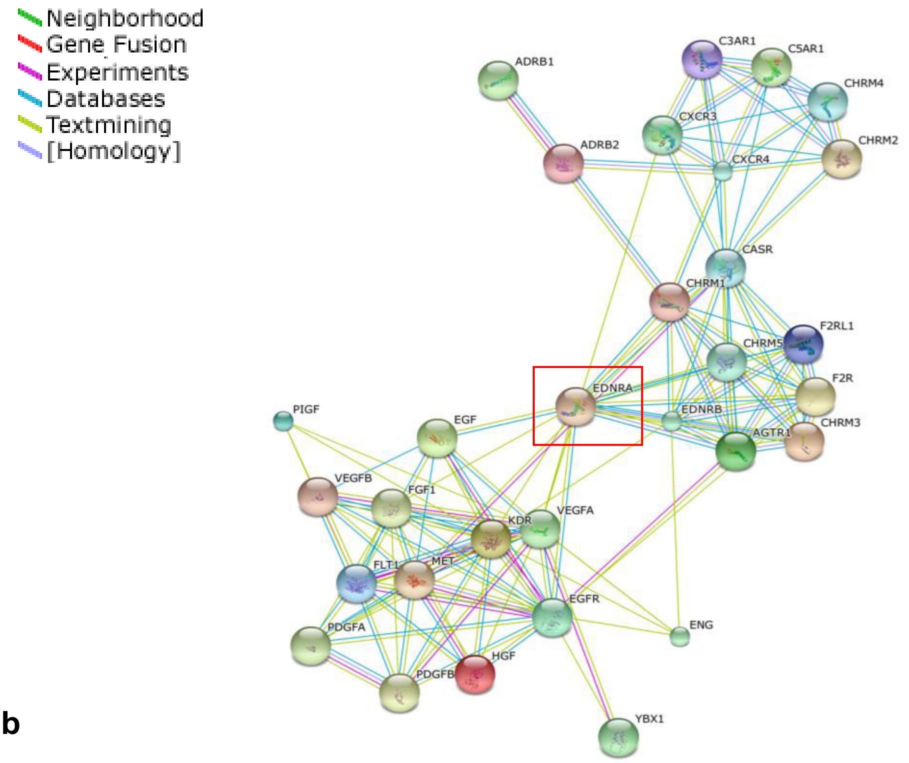


b

| GO Term | GO Biological Process | Molecules | p-value_FDR |
|---|---|---|---|
| **GO:0030334** | regulation of cell migration | VEGFA, HGFR, ENG, VEGFR1, CXCR3, PAR1, VEGFB, CXCR4, C3AR1, HGF, FGF1, EDNRA, VEGFR2, PDGFA, EGFR | $1.26^{-12}$ |
| **GO:0007200** | phospholipase C-activating G-protein coupled receptor signaling pathway | M5, EDNRA, CASR, M1, M3, PAR1, M2, M4, AGTR1 | $1.26^{-12}$ |
| **GO:0040017** | positive regulation of locomotion | VEGFB, HGFR, CASR, VEGFR1, CXCR3, PAR1, C3AR1, HGF, FGF1, EDNRA , VEGFR2, PDGFA, EGFR | $1.26^{-12}$ |

Supplementary Figure 4. Network and gene ontology analysis of autoantibody targets. To help interpret the biological meaning of a putative physiological aab network, we performed gene ontology analysis of aab targets (Extended Data Tab 2, aab dataset 1) using the STRING database. A) Differently colored lines represent different forms of relationship evidence: red lines represent the presence of fusion evidence; green lines show neighborhood evidence; blue lines display cooccurrence evidence; purple lines exhibit experimental evidence; yellow lines demonstrate text mining evidence; and light blue lines display database evidence. The red frame indicates EDNRA in the center of the network. B) The lower panel lists physiological functions regulated by interactions between GPCRs and growth factors or related signaling molecules. Enriched gene ontology (GO) biological processes were considered when the false discovery rate (FDR) was less than 0.05.

## Supplementary Figure 5

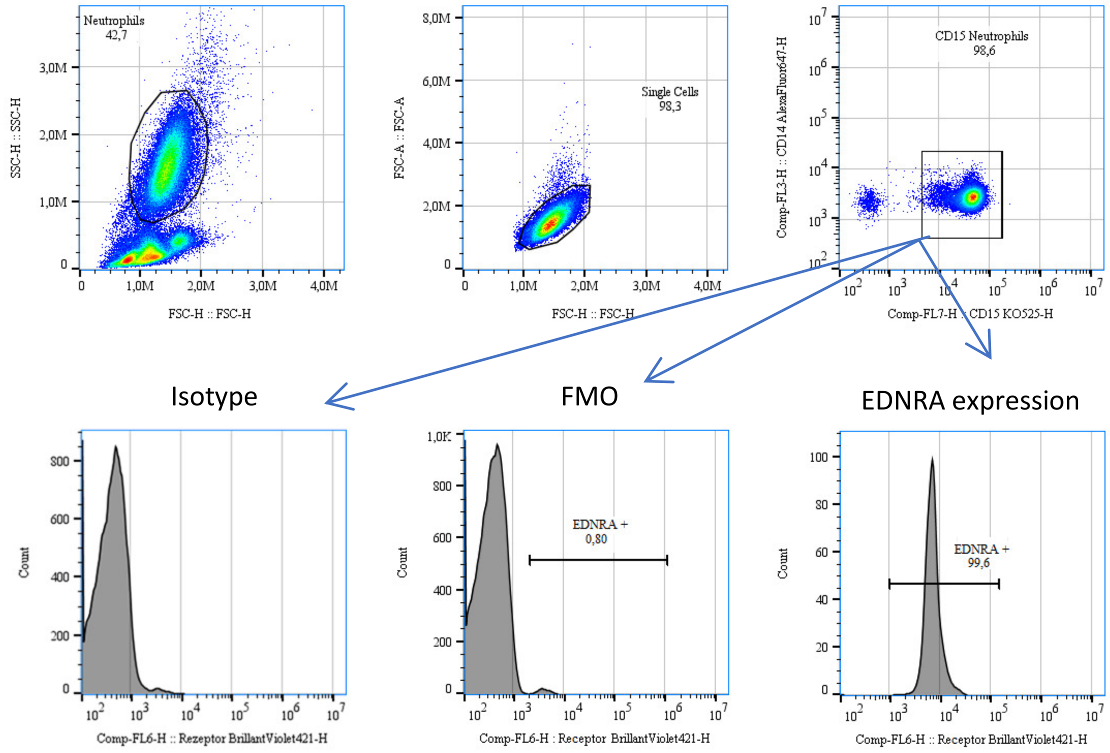


Supplementary Figure 5. Gating strategy for EDNRA expression. For MFI values, an isotype control (Supplementary Table 3) was used to compensate for changes in the cytometry instrument sensitivity. Considering the multiple fluorochromes in the antibody panel to analyze EDNRA expression (Supplementary Table 3), the fluorescence minus one (FMO) control was determined when all the antibodies were present in the flow cytometry tube, except the antibody used to measure EDNRA expression.

**a**

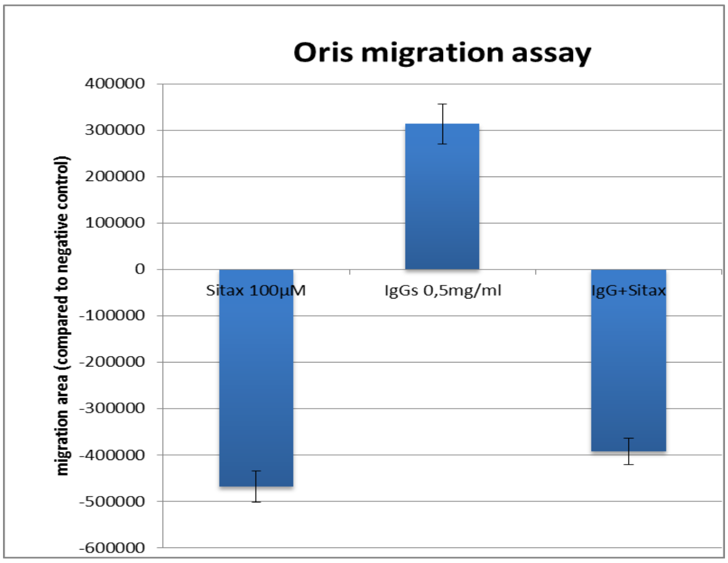


**b**

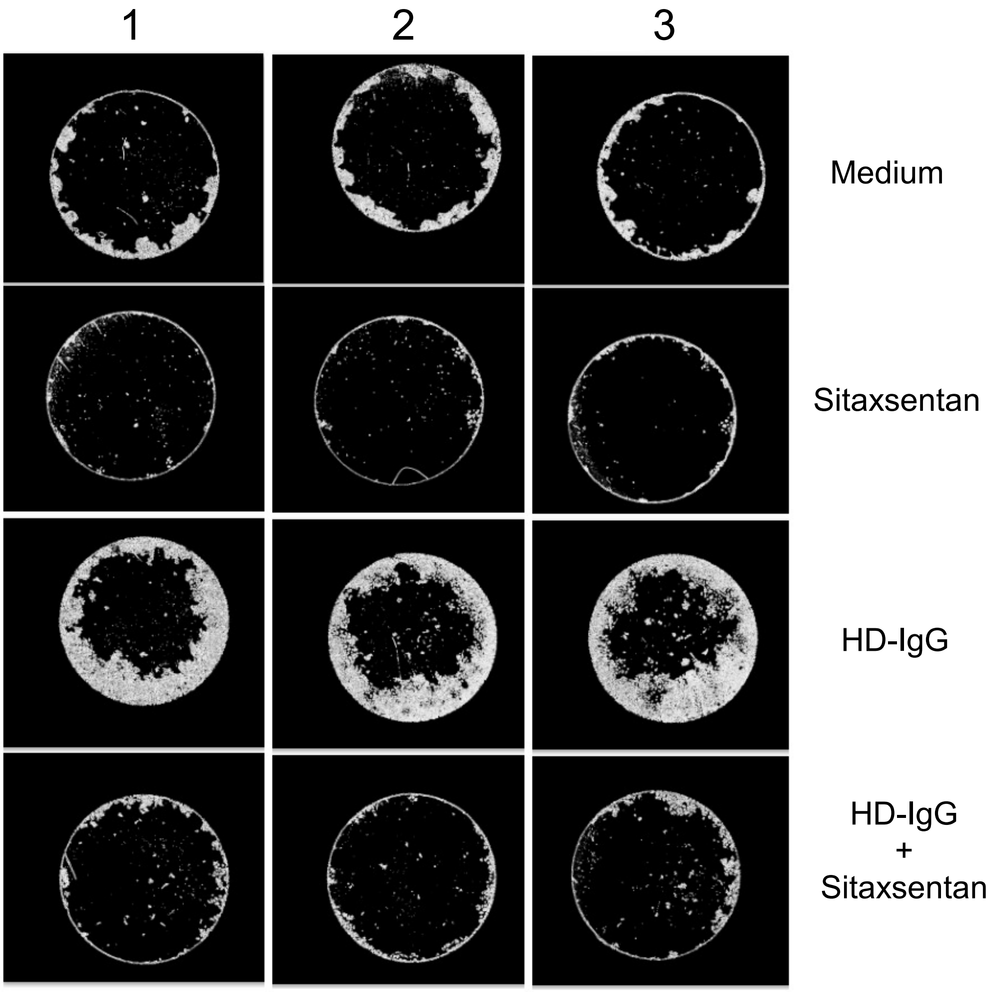


Supplementary Figure 6. Effect of HD-IgG on the migration of the human pancreatic carcinoma Colo357 cell line. The chemotaxis of 3x105 (cells/well) human pancreatic carcinoma Colo357 toward 0.5 mg/ml IgG from healthy donors (HD-IgG) was analyzed using the cell-based Oris™ migration assay. A) The migration area was determined by analyzing B) migration images with the Fiji module of the ImageJ software. Assays were performed in triplicate (1, 2, 3, at the top of the figure). One of three independent experiments (n = 3) is shown. Error bars denote SD. *, $p<0.05$.

**Supplementary Figure 7**

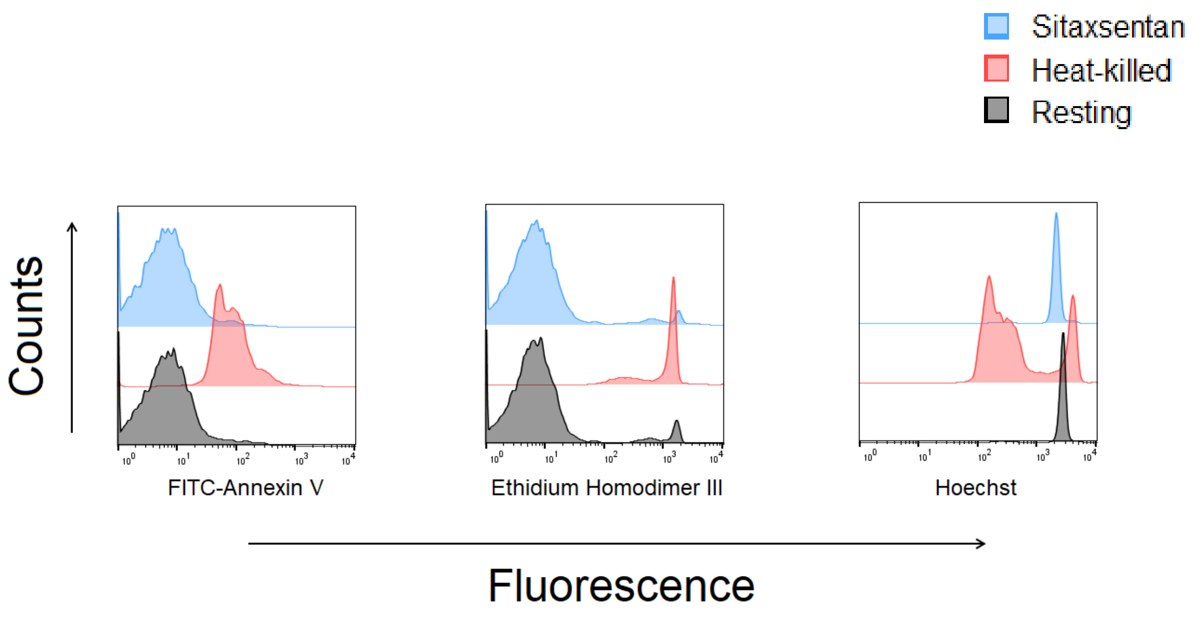


Supplementary Figure 7. Exposure to sitaxsentan, a potent endothelin receptor type A antagonist, has no toxic effect on neutrophils. Neutrophil apoptosis or necrosis was assessed by flow cytometric analysis. The histogram on the left displays apoptotic cells stained by FITC-annexin V; the middle histogram shows necrotic cells stained with ethidium homodimer-III; the histogram on the right demonstrates healthy donor cells stained with Hoechst. Heat-killed cells were used as the experimental control. The results are representative of three independent experiments. The effects of sitaxsentan on neutrophil survival were analyzed by flow cytometry using the Apoptotic/Necrotic/ Healthy Cells Detection Kit (PromoCell, Heidelberg, Germany) according to the manufacturer's instructions.

## Supplementary Figure 8

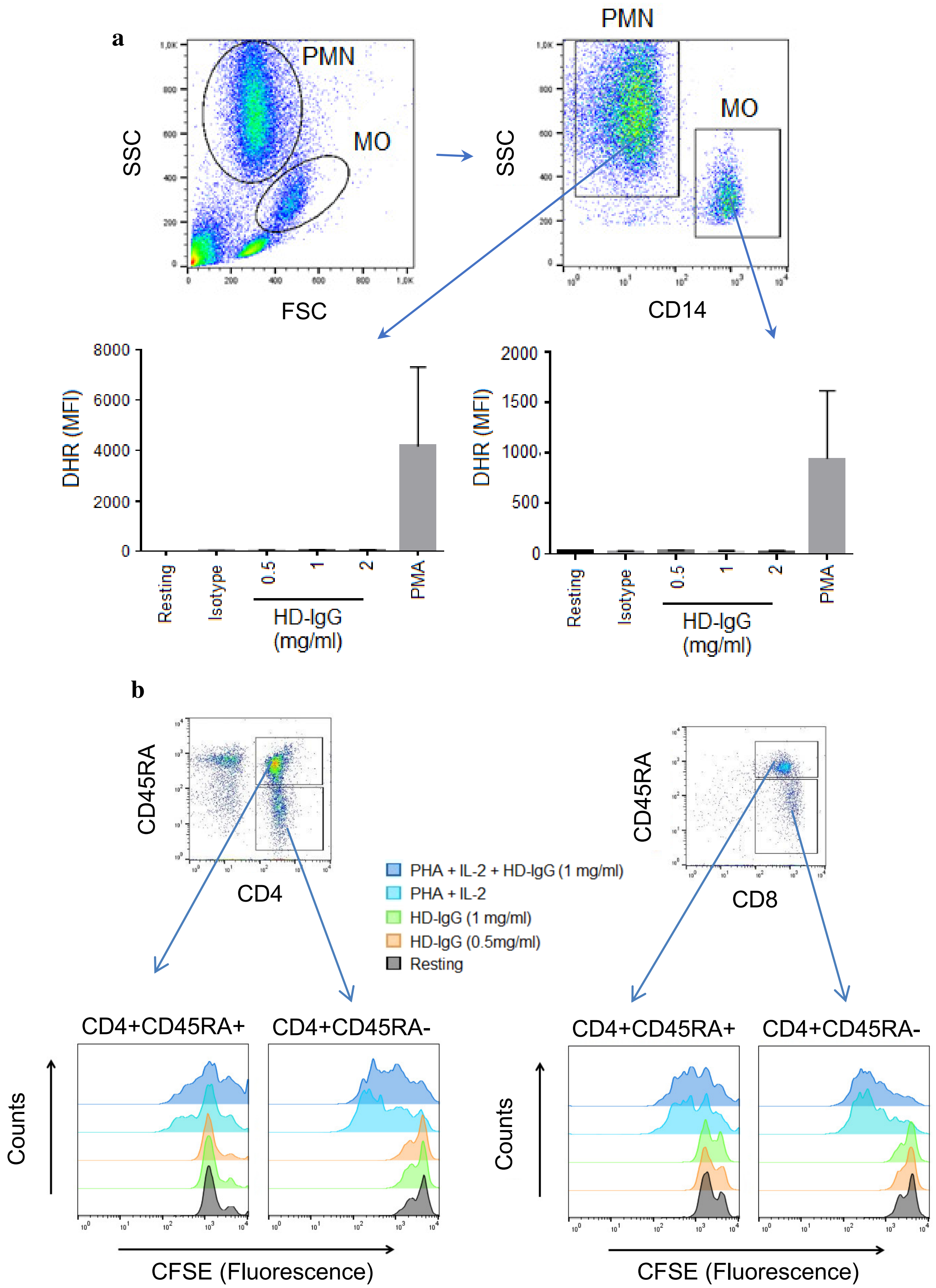


Supplementary Figure 8. Normal human IgG has no effect on the respiratory burst of phagocytes and T cell proliferation. A) The 300 ng/ml phorbol-12-myristate-13-acetate (PMA) but not 0.5 mg/ml healthy donor (HD) IgG induces the respiratory burst of polymorphonuclear neutrophils (PMN) and monocytes (MO). White blood cells were stimulated in vitro in the presence of PMA for 60 min and analyzed by flow cytometry following 400 ng/ml dihydrorhodamine (DHR) 123 staining. Neutrophils and monocytes were gated according to size (forward scatter, FSC), granularity (side scatter, SSC) and pattern of CD14 expression. The median fluorescence intensity (MFI) of the respiratory burst from three different experiments is shown. Error bars denote SD. (B) PBMCs were isolated by Ficoll-Paque density gradient sedimentation. After 5 days at 37°C in the absence or presence of 5 μg/ml phytohemagglutinin (PHA)/10 U/ml of IL-2, robust cell proliferation was observed, but no effect was observed with HD-IgG (n = 3).

**Supplementary Figure 9**

**a**

```
P25101 EDNRA_HUMAN      1    METLCLRASFWLALVGCVISDNPERYSTNLSNHVDDFTTFRGTELSFLVTTHQPTNLVLP    60
Q61614 EDNRA_MOUSE      1    MSIFCLAAYFWLTMVGGVMADNPERYSANLSSHMEDFTPFPGTEINFLGTTHRPPNLALP    60
P21450 EDNRA_BOVIN      1    METFWLRLSFWVALVGGVISDNPESYSTNLSIHVDSVATFHGTELSFVVTTHQPTNLALP    60
Q29010 EDNRA_PIG        1    METFCFRVSFWVALLGCVISDNPESHSTNLSTHVDDFTTFRGTEFSLVVTTHRPTNLALP    60
Q95L55 EDNRA_SHEEP      1    METFWLRVSFWVALVGGVISDNPESYSTNLSIHVDSVTTFRGTELSFVVTTHQPTNLALP    60
W5PE99 W5PE99_SHEEP     1    METFLLRVSFWVALVGGVISDNPESYSTNLSIHVDSVTTFRGTELSFVVTTHQPTNLALP    60
A5A8K3 A5A8K3_RABIT     1    METFCLRASFWLVLIGCVISDNPERYSTNLSNHMDEFTTFHGPELNLLVTTHRPTNLVLP    60
                             *. : :   **:.::* *::**** :*:*** *::..: * * *:.:: ***:* **.**

P25101 EDNRA_HUMAN     61    SNGSMHNYCPQQTKITSAFKYINTVISCTIFIVGMVGNATLLRIIYQNKCMRNGPNALIA   120
Q61614 EDNRA_MOUSE     61    SNGSMHGYCPQQTKITTAFKYINTVISCTIFIVGMVGNATLLRIIYQNKCMRNGPNALIA   120
P21450 EDNRA_BOVIN     61    SNGSMHNYCPQQTKITSAFKYINTVISCTIFIVGMVGNATLLRIIYQNKCMRNGPNALIA   120
Q29010 EDNRA_PIG       61    SNGSMHNYCPQQTKITSAFKYINTVISCTIFIVGMVGNATLLRIIYQNKCMRNGPNALIA   120
Q95L55 EDNRA_SHEEP     61    SNGSMHNYCPQQTKITSAFKYINTVISCTIFIVGMVGNATLLRIIYQNKCMRNGPNALIA   120
W5PE99 W5PE99_SHEEP    61    SNGSMHNYCPQQTKITSAFKYINTVISCTIFIVGMVGNATLLRIIYQNKCMRNGPNALIA   120
A5A8K3 A5A8K3_RABIT    61    SNGSRHNYCPQQTKITSAFKYINTVISCTIFIVGMVGNATLLRIIYQNKCMRNGPNALIA   120
                             **** *.*********.*******************************************

P25101 EDNRA_HUMAN    121    SLALGDLIYVVIDLPINVFKLLAGRWPFDHNDFGVFLCKLFPFLQKSSVGITVLNLCALS   180
Q61614 EDNRA_MOUSE    121    SLALGDLIYVVIDLPINVFKLLAGRWPFDHNDFGVFLCKLFPFLQKSSVGITVLNLCALS   180
P21450 EDNRA_BOVIN    121    SLALGDLIYVVIDLPINVFKLLAGRWPFEQNDFGVFLCKLFPFLQKSSVGITVLNLCALS   180
Q29010 EDNRA_PIG      121    SLALGDLIYVVIDLPINVFKLLAGRWPFENHDFGVFLCKLFPFLQKSSVGITVLNLCALS   180
Q95L55 EDNRA_SHEEP    121    SLALGDLIYVVIDLPINVFKLLAGRWPFEQNDFGVFLCKLFPFLQKSSVGITVLNLCALS   180
W5PE99 W5PE99_SHEEP   121    SLALGDLIYVVIDLPINVFKLLAGRWPFEQNDFGVFLCKLFPFLQKSSVGITVLNLCALS   180
A5A8K3 A5A8K3_RABIT   121    SLALGDLIYVVIDLPINVFKLLAGRWPFDHNDFGVFLCKLFPFLQKSSVGITVLNLCALS   180
                             ****************************:::*****************************

P25101 EDNRA_HUMAN    181    VDRYRAVASWSRVQGIGIPLVTAIEIVSIWILSFILAIPEAIGFVMVPFEYRGEQHKTCM   240
Q61614 EDNRA_MOUSE    181    VDRYRAVASWSRVQGIGIPLITAIEIVSIWILSFILAIPEAIGFVMVPFEYKGELHRTCM   240
P21450 EDNRA_BOVIN    181    VDRYRAVASWSRVQGIGIPLVTAIEIVSIWILSFILAIPEAIGFVMVPFEYKGAQHRTCM   240
Q29010 EDNRA_PIG      181    VDRYRAVASWSRVQGIGIPLVTAIEIVSIWILSFILAIPEAIGFVMVPFEYKGEEHKTCM   240
Q95L55 EDNRA_SHEEP    181    VDRYRAVASWSRVQGIGIPLVTAIEIVSIWILSFILAIPEAIGFVMVPFEYKGAQHRTCM   240
W5PE99 W5PE99_SHEEP   181    VDRYRAVASWSRVQGIGIPLVTAIEIVSIWILSFILAIPEAIGFVMVPFEYKGAQHRTCM   240
A5A8K3 A5A8K3_RABIT   181    VDRYRAVASWSRVQGIGIPLITAIEIVSIWILSFILAIPEAIGFVMVPFEYRGEQHKTCM   240
                             ********************.******************************.*  *.***

P25101 EDNRA_HUMAN    241    LNATSKFMEFYQDVKDWWLFGFYFCMPLVCTAIFYTLMTCEMLNRRNGSLRIALSEHLKQ   300
Q61614 EDNRA_MOUSE    241    LNATSKFMEFYQDVKDWWLFGFYFCMPLVCTAIFYTLMTCEMLNRRNGSLRIALSEHLKQ   300
P21450 EDNRA_BOVIN    241    LNATSKFMEFYQDVKDWWLFGFYFCMPLVCTAIFYTLMTCEMLNRRNGSLRIALSEHLKQ   300
Q29010 EDNRA_PIG      241    LNATSKFMEFYQDVKDWWLFGFYFCMPLVCTAIFYTLMTCEMLNRRNGSLRIALSEHLKQ   300
Q95L55 EDNRA_SHEEP    241    LNATSKFMEFYQDVKDWWLFGFYFCMPLVCTAIFYTLMTCEMLNRRNGSLRIALSEHLKQ   300
W5PE99 W5PE99_SHEEP   241    LNATSKFMEFYQDVKDWWLFGFYFCMPLVCTAIFYTLMTCEMLNRRNGSLRIALSEHLKQ   300
A5A8K3 A5A8K3_RABIT   241    LNATSKFMEFYQDVKDWWLFGFYFCMPLVCTAIFYTLMTCEMLNRRNGSLRIALSEHLKQ   300
                             ************************************************************

P25101 EDNRA_HUMAN    301    RREVAKTVFCLVVIFALCWFPLHLSRILKKTVYNEMDKNRCELLSFLLLMDYIGINLATM   360
Q61614 EDNRA_MOUSE    301    RREVAKTVFCLVVIFALCWFPLHLSRILKKTVYDEMDKNRCELLSFLLLMDYIGINLATM   360
P21450 EDNRA_BOVIN    301    RREVAKTVFCLVVIFALCWFPLHLSRILKKTVYDEMDTNRCELLSFLLLMDYIGINLATM   360
Q29010 EDNRA_PIG      301    RREVAKTVFCLVVIFALCWFPLHLSRILKKTVYDEMDKNRCELLSFLLLMDYIGINLATM   360
Q95L55 EDNRA_SHEEP    301    RREVAKTVFCLVVIFALCWFPLHLSRILKKTVYDEMDTNRCELLSFLLLMDYIGINLATM   360
W5PE99 W5PE99_SHEEP   301    RREVAKTVFCLVVIFALCWFPLHLSRILKKTVYDEMDTNRCELLSFLLLMDYIGINLATM   360
A5A8K3 A5A8K3_RABIT   301    RREVAKTVFCLVVIFALCWFPLHLSRILKKTVYDEMDKNRCELLSFLLLMDYIGINLATM   360
                             *********************************:***.**********************

P25101 EDNRA_HUMAN    361    NSCINPIALYFVSKKFKNCFQSCLCCCCYQSKSLMTSVPMNGTSIQWKNHDQNNHNTDRS   420
Q61614 EDNRA_MOUSE    361    NSCINPIALYFVSKKFKNCFQSCLCCCCHQSKSLMTSVPMNGTSIQWKNQEQNNHNTERS   420
P21450 EDNRA_BOVIN    361    NSCINPIALYFVSKKFKNCFQSCLCCCCYQSKSLMTSVPMNGTSIQWKNHEQNNHNTERS   420
Q29010 EDNRA_PIG      361    NSCINPIALYFVSKKFKNCFQSCLCCCCYQSKSLMTSVPMNGTSIQWKNHEQNNHNTERS   420
Q95L55 EDNRA_SHEEP    361    NSCINPIALYFVSKKFKNCFQSCLCCCCYQSKSLMTSVPMNGTSIQWKNPEQNNHNTERS   420
W5PE99 W5PE99_SHEEP   361    NSCINPIALYFVSKKFKNCFQSCLCCCCYQSKSLMTSVPMNGTSIQWKNPEQNNHNTERS   420
A5A8K3 A5A8K3_RABIT   361    NSCINPIALYFVSKKFKNCFQSCLCCCCHQSKSLMTSVPMNGTSIQWKNHDQNNHNTERS   420
                             ****************************:******************** :******:**

P25101 EDNRA_HUMAN    421    SHKDSMN                                                        427
Q61614 EDNRA_MOUSE    421    SHKDSMN                                                        427
P21450 EDNRA_BOVIN    421    SHKDSIN                                                        427
Q29010 EDNRA_PIG      421    SHKDSIN                                                        427
Q95L55 EDNRA_SHEEP    421    SHKDSIN                                                        427
W5PE99 W5PE99_SHEEP   421    SHKDSIN                                                        427
A5A8K3 A5A8K3_RABIT   421    SHKDSIN                                                        427
                             *****:*
```

**b**

```
P25101 EDNRA_HUMAN    1    METLCLRASFWLALVGCVISDNPERYSTNLSNHVDDFTTFRGTELSFLVTTHQPTNLVLP    60
Q61614 EDNRA_MOUSE    1    MSIFCLAAYFWLTMVGGVMADNPERYSANLSSHMEDFTPFPGTEINFLGTTHRPPNLALP    60
                           *. :** * ***::** *::*******:***.*::*** * ***:.** ***:* **.**

P25101 EDNRA_HUMAN   61    SNGSMHNYCPQQTKITSAFKYINTVISCTIFIVGMVGNATLLRIIYQNKCMRNGPNALIA   120
Q61614 EDNRA_MOUSE   61    SNGSMHGYCPQQTKITTAFKYINTVISCTIFIVGMVGNATLLRIIYQNKCMRNGPNALIA   120
                           ******.*********:*******************************************

P25101 EDNRA_HUMAN  121    SLALGDLIYVVIDLPINVFKLLAGRWPFDHNDFGVFLCKLFPFLQKSSVGITVLNLCALS   180
Q61614 EDNRA_MOUSE  121    SLALGDLIYVVIDLPINVFKLLAGRWPFDHNDFGVFLCKLFPFLQKSSVGITVLNLCALS   180
                           ************************************************************

P25101 EDNRA_HUMAN  181    VDRYRAVASWSRVQGIGIPLVTAIEIVSIWILSFILAIPEAIGFVMVPFEYRGEQHKTCM   240
Q61614 EDNRA_MOUSE  181    VDRYRAVASWSRVQGIGIPLITAIEIVSIWILSFILAIPEAIGFVMVPFEYKGELHRTCM   240
                           ********************:******************************:** *:***

P25101 EDNRA_HUMAN  241    LNATSKFMEFYQDVKDWWLFGFYFCMPLVCTAIFYTLMTCEMLNRRNGSLRIALSEHLKQ   300
Q61614 EDNRA_MOUSE  241    LNATSKFMEFYQDVKDWWLFGFYFCMPLVCTAIFYTLMTCEMLNRRNGSLRIALSEHLKQ   300
                           ************************************************************

P25101 EDNRA_HUMAN  301    RREVAKTVFCLVVIFALCWFPLHLSRILKKTVYNEMDKNRCELLSFLLLMDYIGINLATM   360
Q61614 EDNRA_MOUSE  301    RREVAKTVFCLVVIFALCWFPLHLSRILKKTVYDEMDKNRCELLSFLLLMDYIGINLATM   360
                           *********************************:**************************

P25101 EDNRA_HUMAN  361    NSCINPIALYFVSKKFKNCFQSCLCCCCYQSKSLMTSVPMNGTSIQWKNHDQNNHNTDRS   420
Q61614 EDNRA_MOUSE  361    NSCINPIALYFVSKKFKNCFQSCLCCCCHQSKSLMTSVPMNGTSIQWKNQEQNNHNTERS   420
                           ****************************:********************::******:**

P25101 EDNRA_HUMAN  421    SHKDSMN                                                      427
Q61614 EDNRA_MOUSE  421    SHKDSMN                                                      427
                           *******
```

Supplementary Figure 9. Sequence alignment of endothelin receptor type A. A) A multiple sequence alignment of endothelin receptor type A (EDNRA) shows the high conservation (87.58% identical) among different species and between B) Homo sapiens and Mus musculus (92.27% identical). Alignment of EDNRA was performed using the Clustal Omega program (https://www.UniProt.org/align/) and EDNRA UniProt identifiers.

## Supplementary Table 1

| **HD (cohort 1** | *<65* | *≥65* | *total* | *Mean Age* |
|---|---|---|---|---|
| male | 42 | 24 | 66 | 60.3 ± 7.8 |
| female | 104 | 23 | 127 | 57.6 ± 7.2 |
| *total* | 146 | 47 | 193 | 58.5 ± 7.5 |
| | | | | |
| **Systemic Sclerosis** | *<65* | *≥65* | *total* | *Mean Age* |
| male | 1 | 13 | 14 | 52.8 ± 9.4 |
| female | 62 | 8 | 70 | 56.9 ± 13 |
| *total* | 63 | 21 | 84 | 56.2 ± 12.5 |

| **HD (cohort 2** | *<65* | *≥65* | *total* | *Mean Age* |
|---|---|---|---|---|
| male | 43 | 23 | 66 | 60.4 ± 7.9 |
| female | 104 | 25 | 129 | 57.7 ± 7.1 |
| *total* | 147 | 48 | 195 | 58.9 ± 7.5 |
| | | | | |
| **Ovarian cancer** | *<65* | *≥65* | *total* | *Mean Age* |
| male | 0 | 0 | 0 | 0 |
| female | 141 | 66 | 207 | 59.1 ± 11.4 |
| *total* | 141 | 66 | 207 | 59.1 ± 11.4 |

| **HD (cohort 3** | *<65* | *≥65* | *total* | *Mean Age* |
|---|---|---|---|---|
| male | 1 | 26 | 27 | 73.9 ± 6.5 |
| female | 11 | 65 | 76 | 72.5 ± 8.6 |
| *total* | 12 | 91 | 103 | 73.5 ± 7.5 |
| | | | | |
| **Alzheimer´s disease** | *<65* | *≥65* | *total* | *Mean Age* |
| male | 4 | 21 | 25 | 73.4 ± 7.9 |
| female | 5 | 61 | 66 | 75.8 ± 7.4 |
| *total* | 9 | 82 | 91 | 74.9 ± 7.8 |

Supplementary Table 1. Demographics of healthy donors and patients. All healthy donors (HD) were German subjects not receiving medications known to have any effect on the immune response. Three different HD cohorts, cohort 1 (upper panel), cohort 2 (middle panel) and cohort 3 (lower panel), were used throughout the study for comparison with patients affected by systemic sclerosis (SSc), ovarian cancer (OC) and Alzheimer's disease (AD), respectively.

# Supplementary Table 2

| Aab dataset 1 – HD cohort 1 and SSc | Full Name |
|---|---|
| *G protein-coupled receptors* | |
| **AT1R or *AGTR1*** | angiotensin II receptor type 1 |
| **ADRB1** | beta-1 adrenergic receptor |
| **ADRB2** | beta-2 adrenergic receptor |
| **CASR** | calcium-sensing receptor |
| **CXCR3** | chemokine (C-X-C motif receptor 3 |
| **CXCR4** | chemokine (C-X-C motif receptor 4 |
| **C3AR1** | complement component 3a receptor 1 |
| **C5AR1** | complement component 5a receptor 1 |
| **ETAR or *EDNRA*** | endothelin receptor type A |
| **ETBR *EDNRB*** | endothelin receptor type B |
| **M1 or *CHRM1*** | cholinergic receptor muscarinic 1 |
| **M2 or *CHRM2*** | cholinergic receptor muscarinic 2 |
| **M3 or *CHRM3*** | cholinergic receptor muscarinic 3 |
| **M4 or *CHRM4*** | cholinergic receptor muscarinic 4 |
| **M5 or *CHRM5*** | cholinergic receptor muscarinic 5 |
| **PAR1 or *F2R*** | Protease-activated receptor 1 |
| **PAR2 or *F2RL1*** | Protease-activated receptor 2 |
| *Growth factors* | |
| **EGF** | epidermal growth factor |
| **FGF1** | fibroblast growth factor 1 |
| **HGF** | hepatocyte growth factor |
| **PDGFA** | platelet-derived growth factor alpha |
| **PDGFB** | platelet-derived growth factor beta |
| **VEGFA** | vascular endothelial growth factor A |
| **VEGFB** | vascular endothelial growth factor B |
| **PIGF** | Placental growth factor |
| *Growth factor receptors* | |
| **EGFR** | epidermal growth factor receptor |
| **HGFR or *MET*** | hepatocyte growth factor receptor |
| **VEGFR1 or *FLT1*** | vascular endothelial growth factor 1 |
| **VEGFR2 or *KDR*** | vascular endothelial growth factor 2 |
| *Signaling molecules* | |
| **YBX1** | Y-box-binding protein 1 |
| **ENG** | Endoglin |

| Aab dataset 2 - HD cohort 2 and OC | Full Name |
|---|---|
| *G protein-coupled receptors* | |
| **AT1R or *AGTR1*** | angiotensin II receptor type 1 |
| **CASR** | calcium-sensing receptor |
| **CXCR3** | chemokine (C-X-C motif receptor 3 |
| **CXCR4** | chemokine (C-X-C motif receptor 4 |
| **ETAR or *EDNRA*** | endothelin receptor type A |
| **ETBR *EDNRB*** | endothelin receptor type B |
| **PAR1 or *F2R*** | Protease-activated receptor 1 |
| **PAR2 or *F2RL1*** | Protease-activated receptor 2 |
| *Growth factor receptors* | |
| **EGF** | epidermal growth factor |
| **FGF1** | fibroblast growth factor |
| **HGF** | hepatocyte growth factor |
| **PDGFA** | platelet-derived growth factor alpha |
| **PDGFB** | platelet-derived growth factor beta |
| **VEGFA** | vascular endothelial growth factor A |
| **VEGFB** | vascular endothelial growth factor B |
| **PIGF** | Placental growth factor |
| *Growth factor receptors* | |
| **EGFR** | epidermal growth factor receptor |
| **HGFR or *MET*** | hepatocyte growth factor receptor |
| **VEGFR1 or *FLT1*** | vascular endothelial growth factor 1 |
| **VEGFR2 or *KDR*** | vascular endothelial growth factor 2 |
| *Signaling molecules* | |
| **YBX1** | Y-box-binding protein 1 |
| *Scavenger receptors* | |
| **STAB1** | Stabilin-1 |
| **STAB2** | Stabilin-2 |

| Aab dataset 3 – HD cohort 3 and AD | Full Name |
|---|---|
| *G protein-coupled receptors* | |
| **ADRB1** | beta-1 adrenergic receptor |
| **ADRB2** | beta-2 adrenergic receptor |
| **C5AR1** | complement component 5a receptor 1 |
| **ETAR or *EDNRA*** | endothelin receptor type A |
| **M1 or *CHRM1*** | cholinergic receptor muscarinic 1 |
| **M2 or *CHRM2*** | cholinergic receptor muscarinic 2 |
| **M3 or *CHRM3*** | cholinergic receptor muscarinic 3 |
| **M4 or *CHRM4*** | cholinergic receptor muscarinic 4 |
| **M5 or *CHRM5*** | cholinergic receptor muscarinic 5 |
| **PAR1 or *F2R*** | Protease-activated receptor 1 |
| *Growth factors* | |
| **PDGFA** | platelet-derived growth factor alpha polypeptide |
| **VEGFA** | vascular endothelial growth factor A |
| *Growth factor receptors* | |
| **VEGFR1 or *FLT1*** | vascular endothelial growth factor 1 |
| **VEGFR2 or *KDR*** | vascular endothelial growth factor 2 |
| *Neurological or AD-associated Molecules* | |
| **$\alpha_1$AR** | Alpha 1 adrenoceptor |
| **α2AR** | Alpha 2 adrenoceptor |
| **D1R** | D1 Dopamine receptor |
| **D2SR** | D2s Dopamine receptor |
| **D3R** | D3 Dopamine receptor |
| **D42R** | D42 Dopamine receptor |
| **D44R** | D44 Dopamine receptor |
| **D47R** | D47 Dopamine receptor |
| **HT1AR** | 5-hydroxytryptamine receptor 1 |
| **HT2AR** | 5-hydroxytryptamine receptor 2A |
| **HT2BR** | 5-hydroxytryptamine receptor 2B |
| **HT2CR** | 5-hydroxytryptamine receptor 2C |
| **HT5AR** | 5-hydroxytryptamine receptor 5 |
| **HT6R** | 5-hydroxytryptamine receptor 6 |
| **HT7R** | 5-hydroxytryptamine receptor 7 |
| **NGF** | Nerve growth factor beta |
| **RAGE** | Receptor for advanced glycation end products |
| *Signaling molecules* | |
| **YBX1** | Y-box-binding protein 1 |
| **ENG** | Endoglin |
| *Scavenger receptors* | |
| **STAB1** | Stabilin-1 |
| **STAB2** | Stabilin-2 |

Supplementary Table 2. Autoantibody datasets. Three datasets of autoantibodies (aab) were analyzed in sera from three healthy donor (HD) cohorts and patients with systemic sclerosis (SSc, upper left panel), ovarian cancer (OC, lower left panel) and Alzheimer's disease (AD, upper right panel).

## Supplementary Table 3

| **Antibodies** | | | | |
|---|---|---|---|---|
| *Antibody targets* | *Fluorochrome* | *Dilution* | *Clone or number* | *Manufacturer* |
| CD14 | PE-Cy7/Alexa Fluor 647 | 1:200/1:50 | HCD14/ M5E2 | Biolegend |
| CD15 | Brillant Violet 510 | 1:50 | W6D3 | Biolegend |
| Anti-Rabbit IgG | CFL405 | 1:100 | Sc-362252 | SantaCruz |
| Anti-Rabbit IgG | Brillant Violet 421 | 1:500 | Poly4064 | Biolegend |
| EDNRA | n.a. | 1:100 | sc-33535 | Santa Cruz |
| Isotype 1 | n.a. | 1:100 | sc-3888 | Santa Cruz |

Supplementary Table 3. Antibody panel used for flow cytometric analyses of EDNRA expression.
